# Evaluation of mesoporous silicon thermal conductivity by electrothermal finite element simulation

**DOI:** 10.1186/1556-276X-7-427

**Published:** 2012-07-31

**Authors:** Laurent Siegert, Marie Capelle, Fabrice Roqueta, Vladimir Lysenko, Gael Gautier

**Affiliations:** 1 Laboratoire de Microélectronique de Puissance, GREMAN, Université de Tours, 16 rue Pierre et Marie Curie, BP 7155, Tours, 37071, France; 2 STMicroelectronics Tours R&D, 16 rue Pierre et Marie Curie, Tours Cedex, 37071, France; 3 Institut des Nanotechnologies de Lyon, Domaine Scientifique de la Doua, INSA de Lyon, Bat. Blaise Pascal, 7 Av. J. Capelle, Villeurbanne Cedex, F-69621, France

**Keywords:** Mesoporous silicon, Thermal conductivity, Electrothermal simulation, Calibration

## Abstract

The aim of this work is to determine the thermal conductivity of mesoporous silicon (PoSi) by fitting the experimental results with simulated ones. The electrothermal response (resistance versus applied current) of differently designed test lines integrated onto PoSi/silicon substrates and the bulk were compared to the simulations. The PoSi thermal conductivity was the single parameter used to fit the experimental results. The obtained thermal conductivity values were compared with those determined from Raman scattering measurements, and a good agreement between both methods was found. This methodology can be used to easily determine the thermal conductivity value for various porous silicon morphologies.

## Background

Application of porous silicon for electrical isolation in radiofrequency devices is widely studied [1,2]. However, characterization of its thermal properties is very important, too, in order to anticipate any thermal risk due to self-heating effects. Thermal conductivity of porous silicon depends mainly on its own morphological parameters at nanoscale level, such as pore morphology and average size of Si nanocrystallites [3].

There are several existing methods to measure thermal conductivity which include scanning thermal microscopy, that maps the local temperature and thermal conductivity of an interface [4], the 3*ω* method, which determines the thermal conductivity by applying an AC signal to metal strip [5], or the modeling approach [6], which is used in modeling the effect of pore size, pore arrangement, and porosity. The principle of comparing electrothermal simulations and experiments in order to determine thermal parameters of a material has already been applied successfully [7,8].

In this study, we propose to apply this method in order to evaluate the thermal conductivity of a porous silicon nanomaterial (mesoporous in our case) by electrical characterization of metal strips with subsequent electrothermal finite element simulations. These metal strips are integrated with the dielectric layers on top of bulk silicon and mesoporous silicon PoSi/silicon substrates. The electrical tests have been performed with a probe station on various test lines with different widths of 10, 30, 50 μm and a constant length of 1,100 μm. The electrical resistance of the test line is measured versus the applied current. This resistance is not constant with the current and varies due to the resistance temperature dependence. Firstly, the model parameters have been calibrated by comparing experimental and simulation results for test lines integrated onto bulk silicon substrate. Secondly, thermal conductivity of the mesoporous silicon has been determined by fitting the experimental and simulation results for test lines integrated onto PoSi/Si substrate. To validate this approach, a micro-Raman thermal conductivity measurement is performed.

## Methods

### Silicon anodization

Six inch porous silicon samples were fabricated by anodization of highly *n*-doped (100) silicon wafers *(ρ*  = 10 mΩ·cm). The anodization was run in a HF (50%)-acetic acid-water solution (4.63:2.14:1.43). Acetic acid has been used as a surfactant which improves the electrolyte penetration into the pores [9]. Moreover, acetic acid is known to improve the roughness and the uniformity of porous layers as well as to increase the etch rate [10].

**Figure 1:**
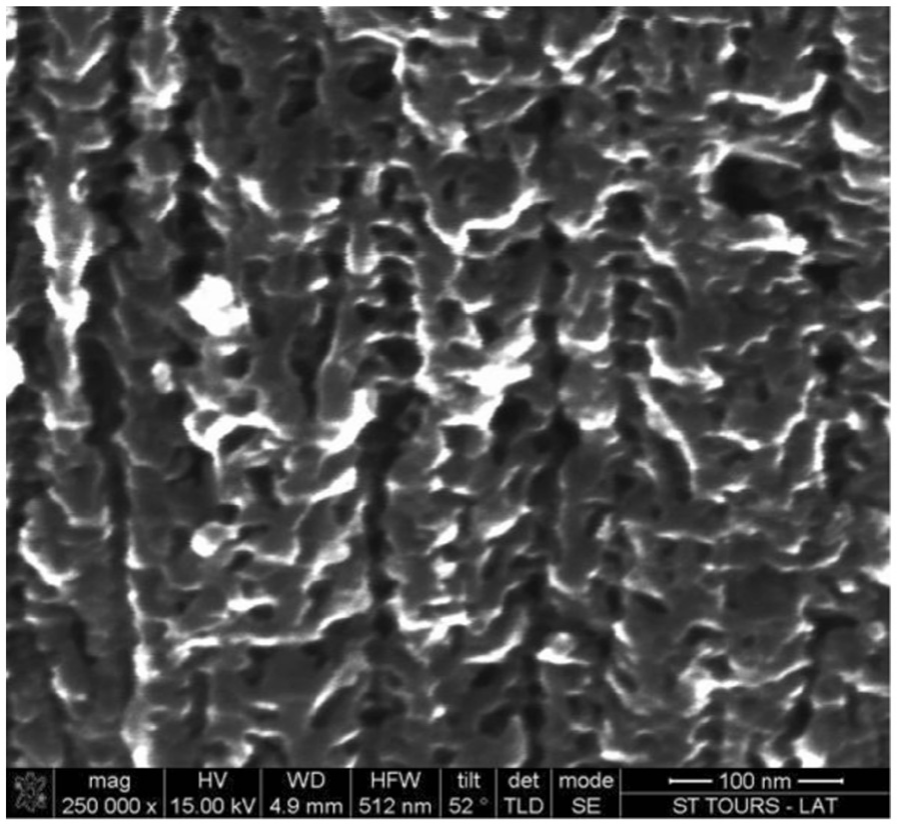
**Cross-sectional SEM image of typical mesoporous silicon.** This is fabricated by the anodization of a highly *n*-doped silicon substrate in HF-acetic acid-water solution.

Anodization was performed in a double tank electrochemical cell. A mesoporous layer 100-μm thick was obtained by 60 min of anodization at 14.5 mA/cm². Mesoporous thicknesses were measured by scanning electron microscopy (SEM) after splitting the samples. An average 20% porosity was evaluated by weight measurements. Finally, porous silicon samples have been annealed at 300°C under N_2_ for 1 h in order to stabilize the structure. The typical morphology observed in our case is presented in Figure 1.

### Test line patterns

Test lines with various designs were integrated on the porous silicon samples of highly *n*-doped silicon (*ρ*  = 10 mΩ·cm). A 500-nm plasma enhanced chemical vapor deposition (PECVD) oxide has been deposited as a cap layer on the porous silicon. Test lines were made with 1-μm aluminum deposited at 350°C by physical vapor deposition (PVD). A 500-nm PECVD oxide was then deposited to passivate the aluminum. Finally, the electrical contact to the first metal level has been made with 1-μm PVD aluminum. The patterns were defined by standard photolithography and dry etching with chloride-based plasma by reactive ion etching.

**Figure 2:**
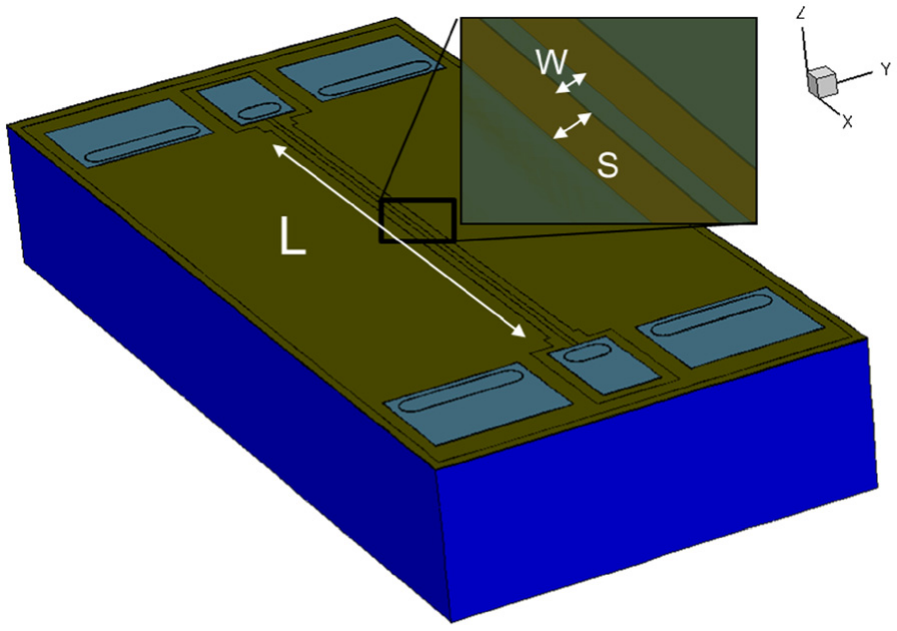
**Three-dimensional structure for simulation.** Aluminum (clear blue) is embedded by the oxide (green) on a silicon substrate (dark blue).

In this work, test lines of 10-, 30-, 50-μm strip widths (*W*) were fabricated. The length (*L*) and the spacing to the surrounding ground plane (*S*) are set to 1,100 and 15 μm, respectively. For instance, a structure with 1,100 μm in length, 10 μm in width, and 15 μm of spacing to the ground plane is denoted as *W*10*S*15*L* 1100 as shown in Figure 2.

## Results and discussion

### Results

#### Electrical test

These test lines are electrically characterized by the application of a current stress; the experiments have been done with a probe station by four wire sensing measurements. The resistance of a test line is measured versus the applied current. The resistance is not constant with the current but varies due to temperature dependence. Resistance (or resistivity) varies with temperature following a linear law:

(1) $$R(T)=R_{0}.(1+{\text{TCR}}_{0}.T)$$

where *R*(*T*) is the resistance depending to the temperature; *R*_0_, the resistance at 0°C; and TCR_0_, the temperature coefficient of resistance at 0°C.

**Figure 3:**
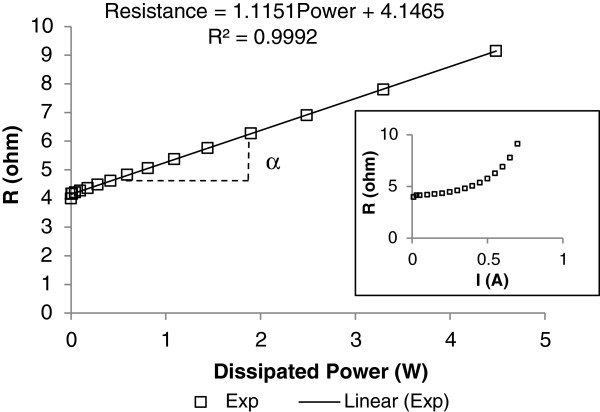
**The resistance varies with the applied current (enclosed).** The resistance follows a linear variation with the dissipated power. The slope is defined as the alpha coefficient.

Under a current stress, the test line heats, and its resistance changes, proportional to the temperature rise. This resistance variation is linear versus the dissipated power (*RI*²) as shown in Figure 3. The alpha coefficient is defined as the slope and is typical to the Joule heating effect.

For each structure on silicon substrate and porous Si/silicon substrate, the resistance rise is measured, and the alpha coefficient is calculated by a linear regression. Experimental results are given in Table 1.

**Table 1:** Alpha coefficient calculation for the different dimension strips for both substrates (Si and PoSi)

	**Alpha (Ω·W**^**−1**^**)**	
**Dimension strip**	**Substrate**	
	**Si**	**PoSi/Si**
W10	1.115	4.61
W30	0.199	0.93
W50	0.09	0.502

#### Electrothermal simulation

The simulator used is the Sentaurus Sdevice tool from Synopsis, Inc. (Mountain View, CA, USA). The structure is created and meshed. Electrothermal simulation is then performed. The model uses Poisson's equation coupled with temperature. The temperature induced by the current is calculated, and the dissipation is computed by using Fourier's law. Both effects are calculated at the same time (coupled simulation).

This model depends on many parameters necessary to be implemented in order to make the simulation reliable.

In order to model the experiments, different parameters (dimensional, material properties, boundaries conditions) have to be investigated, determined, and implemented in the simulation. Electrothermal simulation requires a good knowledge of the materials' properties; for this reason, the model simulation has to be calibrated.

· *Aluminum parameters*. The aluminum parameters are defined as follows: the dimensions (width and thickness), the resistivity at 0°C, and the TCR at 0°C. TCR is determined as 0.0045°C^−1^ by the following measurement. A temperature ramp is applied to several patterns (NIST), and their ohmic resistances are measured at low current (to avoid joule heating). A straight line is obtained, and the TCR is defined as the slope divided by the resistance at a given temperature. Finally, an average value of TCR is obtained. The aluminum thickness is determined by means of a focused ion beam (FIB) cut and a SEM measurement. The resistivity (*ρ*) is calculated by taking the resistance measurement at low current (with no joule heating influence), the thickness measurement, and the theoretical width. Calculation is performed for each pattern using Equation 2:

(2) $$\rho (30{}^{\circ}\text{C})=R(30{}^{\circ}\text{C})\frac{T\times W}{L}$$

Results are given in Table 2. The aluminum resistivity is almost constant, around 3.30 × 10^−8^ Ω·m; the resistivity at 0°C is then calculated with Equation 3:

(3) $$\rho_{0}=\frac{\rho (T)}{\left(1+{\text{TCR}}_{0}\cdot \text{T}\right)}$$

with *T* = 30°C by taking the TCR defined as above.

**Table 2:** Dimensions (μm), resistance (Ω), and resistivity (Ω·m) of the three patterns

	**Th (μm)**	***W*(μm)**	***L*(μm)**	***R*(Ω) at 30°C**	***ρ*(Ω·m) at 30°C**
W10	0.94	9.4	1,100	4.053	3.26 × 10^−8^
W30	0.94	29.5	1,100	1.307	3.30 × 10^−8^
W50	0.94	49	1,100	0.796	3.33 × 10^−8^

*Th*, thickness; *W*, width; *L*, length. From the resistance (*R*) measurement, resistivity (*ρ*) is calculated.

· *Stack layer parameters: thermal conductivity and thickness*. The thermal conductivity coupled with the thickness determines the thermal resistance and the dissipation. The oxide thickness is determined by the FIB cut. Thermal conductivity is taken according to the literature and equal to 1.4 W/(K·m)[11]. Nevertheless, this value can change depending on thermal contact resistance between the oxide and the substrate. For the silicon substrate, the thermal conductivity (λ_Si_) is given by Equation 4 [12]:

(4) $$\lambda_{\mathrm{Si}}(T)=\frac{1}{\left(a+b.T+cT^{2}\right)}$$

with *a*  = 0.03 K·m·W^−1^*b* = 1.56 × 10^−3^ m·W^−1^, and *c* = 1.65 × 10^−6^ m·K^−1^·W^−1^.

The silicon thermal conductivity (TC) varies with a number of parameters such as impurities, doping level, and carrier concentration. In this work, we have taken the general equation of silicon TC and make the assumption that the difference between both values was too weak to influence the final result (metal heating).

· *Boundary conditions*. The meaning of the boundary conditions (BC) is to replicate the experimental setup [13]. There are two kinds of BCs: thermal and electrical. The input of the electrical BC is rather trivial; it is just a reference potential, and the input/output terminal (electrical contact) has to be set. The specification of the thermal BCs, however, is more complex. Two thermal conditions may be defined: at the top surface, to take into accounts the thermal exchange with air, and at the bottom surface, at the chuck/substrate interface. The chuck temperature is at 30°C; this temperature is applied to the bottom surface. At the top surface, there is natural convection with air. We use the traditional value of natural convection of 26 W/(K·m²).

### Model simulator calibration on silicon substrate

**Figure 4:**
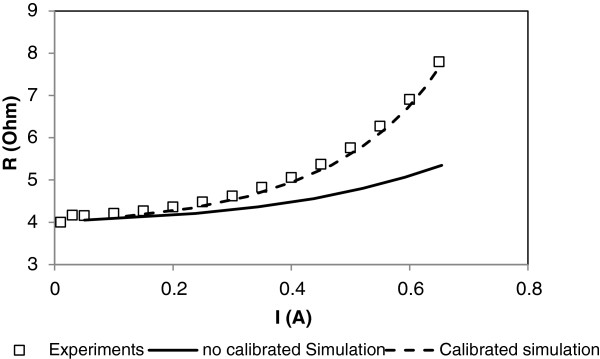
**Comparison between experiments (empty squares) W10S15L1100 and simulation before (line) and after (broken line) calibration.** Before the calibration, a mismatch between the simulated response and the experiments is found. This mismatch is corrected after the determination of the models' parameters.

An initial simulation is performed with implemented values; from the simulation, the resistance versus the current are plotted and compared to the experiments as shown in Figure 4 (full line).

Two conclusions can be made. Firstly, values of simulated resistance and experimental resistances are matched at low current. This means that electrical and dimensional parameters for the aluminum are correct. Secondly, for the high current, the simulation diverges from experiments. Simulated DC resistance evolution is lower than the experimental DC resistance. The simulation computes a higher dissipation of the heat generated in the line than what actually occurs in the experiments.

**Figure 5:**
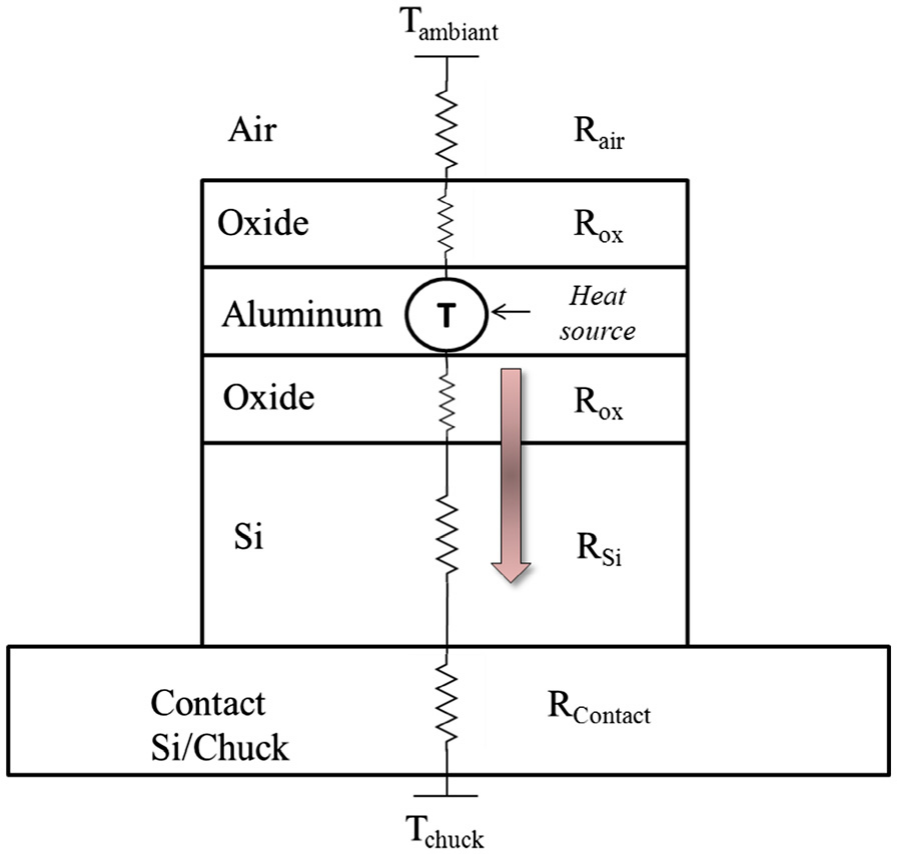
**Thermal path for our structure.** The main heat path is through the substrate.

Considering the simplified heat path through the structure given in Figure 5, two major parameters can be chosen to match the simulation to the experimental results. The main path starts from the line through the oxide layer down to the silicon and afterward to the chuck. Considering the thermal conductivity of silicon which is much larger than that of the oxide, the oxide thermal conductivity is chosen in order to fit the experiments. Another parameter, BC at the bottom surface, supposed a perfect contact between the chuck and the silicon substrates. In reality, there are some imperfections which existed called ‘contact resistance’. In order to take into account this imperfection, a heat coefficient is set at this interface.

Two parameters have been chosen: the thermal conductivity of the oxide and the BC at the bottom surface. The goal is to vary these parameters in the simulation in order to reach a fit of experimental data. A design experiment is performed on these two parameters defined as the factors. The response is the alpha coefficient. Simulations are performed for each pattern. This makes a total of 27 simulations (3 × 9).

Finally, having both values unknown, the heat film coefficient and the oxide thermal conductivity are found to be 3.4 W/(K·m²) and 1.1 W/(K·m), respectively. We have noticed that this last value is not far from the oxide thermal conductivity found in the literature, which is from 1.0 to 1.2 W/(K·m) for a thin silicon dioxide film [14]. Simulations were performed for each pattern with these values in order to check their validity (Figure 4 broken line). The comparison of experimental and simulated alpha coefficients is given in Table 3. 

**Table 3:** Comparison of the alpha coefficients between the experiments and the simulations after calibration

	***α*Experimental (Ω·W**^**−1**^**)**	***α*Simulated (Ω·W**^**−1**^**)**	**Difference (%)**
W10	1.115	1.048	5.98
W30	0.199	0.180	9.33
W50	0.09	0.092	2.57

The parameters found and the conditions in the probe station are fixed and used for the PoSi thermal conductivity determination.

### PoSi thermal conductivity determination

**Figure 6:**
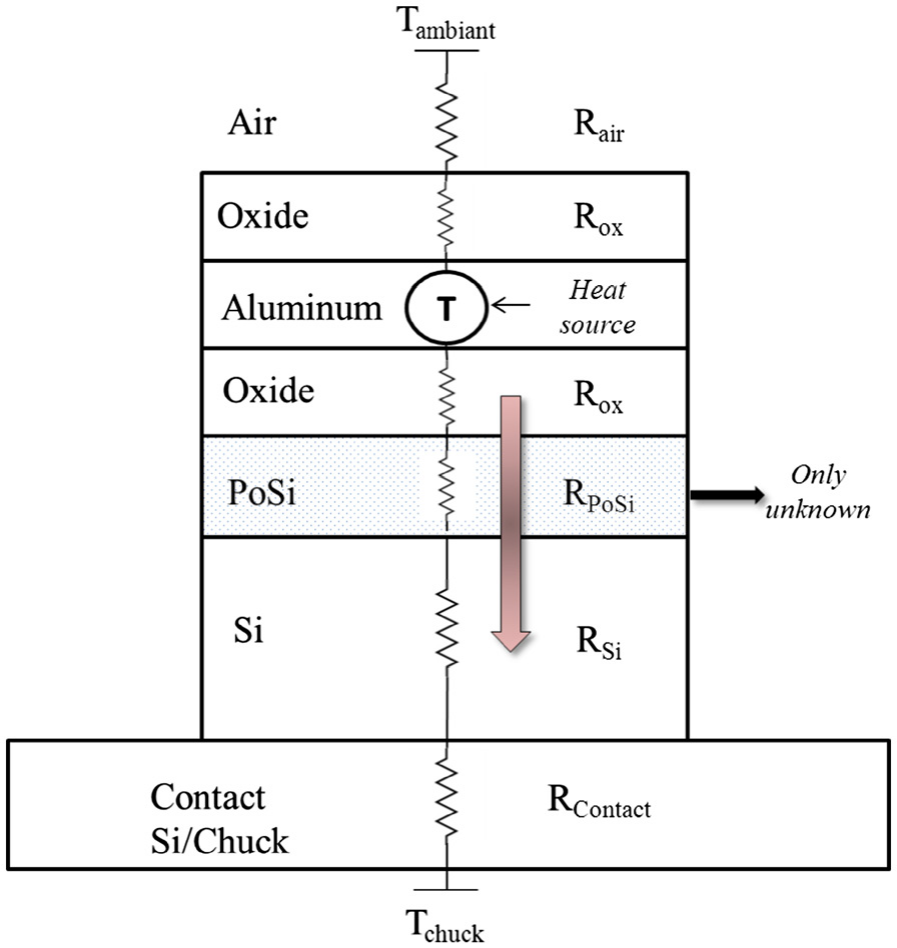
Thermal path for our structure with a PoSi layer.

With this step, the model simulator is calibrated for our experiments (prober station electrical test). The stack described previously is now integrated on a 100-μm PoSi layer. Therefore, the only unknown is the thermal conductivity of this new layer in the stack (Figure 6).

The thermal conductivity of the porous silicon is largely dependent on many parameters, intrinsically to porous silicon: pore size, depth, density, substrate P or N [15].

Electrical tests are performed on the same test line integrated with PoSi. The same methodology is used as described above: the resistance versus current is measured; the resistance versus dissipated power is plotted; and then the alpha coefficient (slope) is computed.

**Figure 7:**
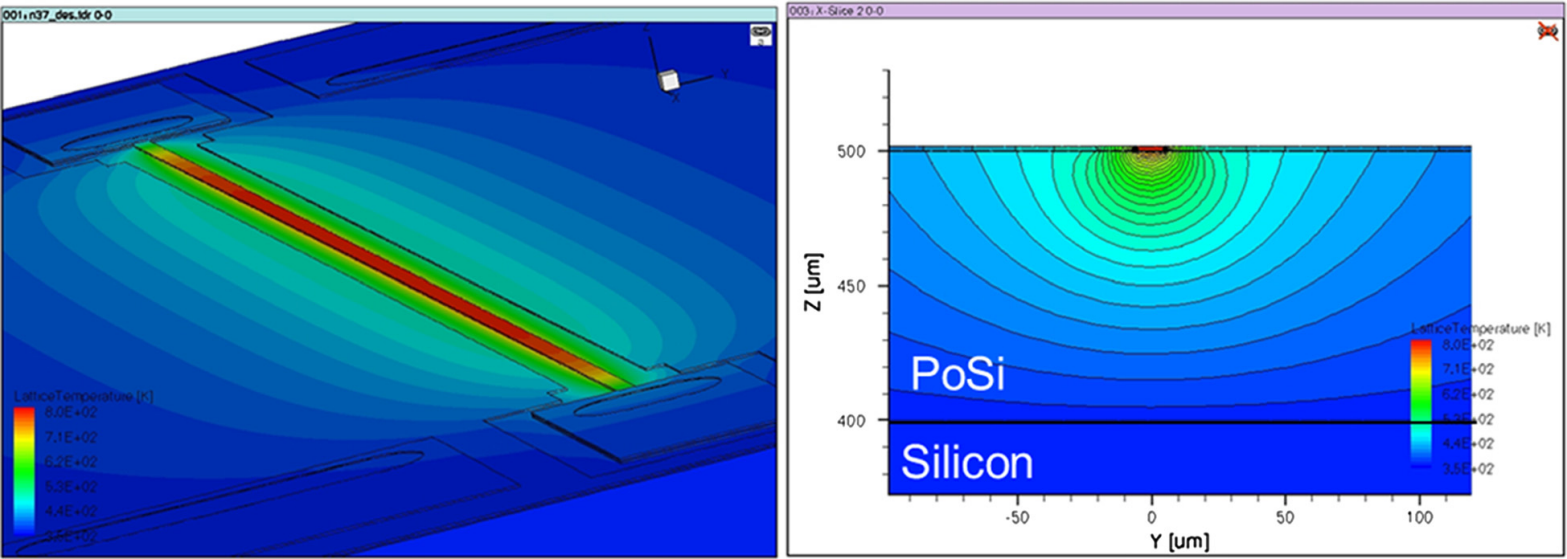
**Electrothermal simulation of W10S15L1100 on porous silicon/silicon substrate.** The porous silicon is simulated by a material layer. Thermal conductivity of this layer varies until reaching agreements with experiments.

The value of the mesoporous silicon (our case) thermal conductivity varies from 1 to 7 W/(K·m) in literature [16]. In the simulation, a virtual layer is added with a variable thermal conductivity (Figure 7); we use three levels of variation: 3, 5, and 7 W/(K·m). 

**Figure 8:**
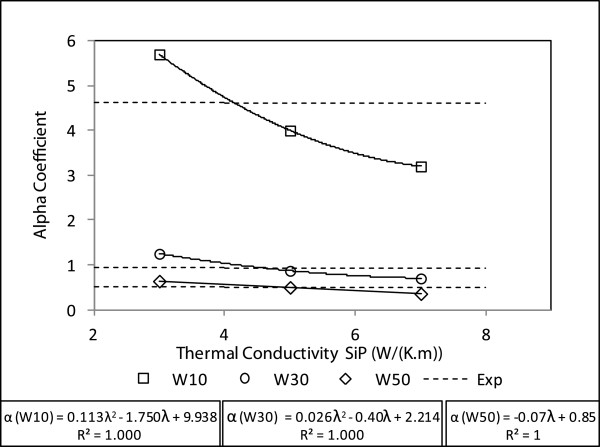
**Alpha coefficient for different thermal conductivities (dot) versus the porous silicon thermal conductivity set in simulation.** From this graph, a porous silicon thermal conductivity value is found according to experimental alpha coefficient.

For each thermal conductivity, the alpha coefficient is calculated. The results for each pattern are given in Figure 8.

By multi-response optimization, a thermal conductivity is deduced to fit the experimental alpha value for each pattern. The best results (with the lowest error with experimental values) give 4.2 W/(K·m).

**Figure 9:**
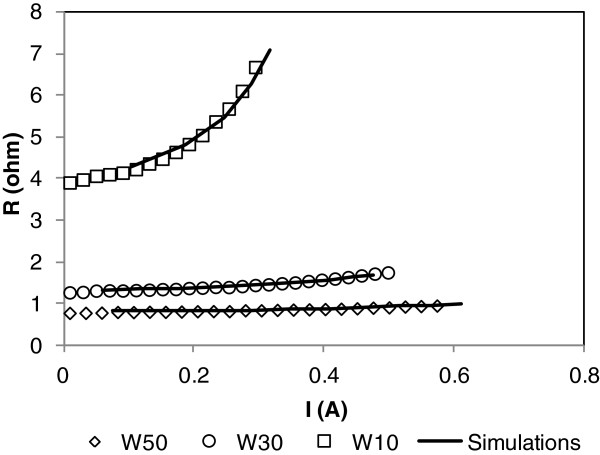
**Simulations performed for each dimension (W10, W30, W50).** Simulations fit experiments validating the thermal conductivity value determined previously.

To check the determined thermal conductivity value, last simulations are performed for each pattern; results are given in Figure 9. A good prediction of the resistance rise versus the current is found.

### Thermal conductivity micro-Raman measurement

Micro-Raman scattering spectrometry [17] has been used to measure directly thermal conductivity value of our porous Si layers. From this measurement, we have obtained a value of 5 W/(K·m), confirming the validity of our simulation methodology.

The difference between these methodologies can explain the small gap between both values.

### Discussion

In this work, we have evaluated by electrical measurement coupled with electrothermal simulation the mesoporous silicon thermal conductivity. In order to validate the determined value, we also performed a micro-Raman thermal conductivity measurement. We found a good agreement between both values.

In the model, some assumptions have been taken: mesoporous silicon is assumed to be a bulk material. The pore size and the morphology are not considered. Therefore, the thermal-determined conductivity value is an average value.

Moreover, to apply this methodology, the thermal conductivity value of the PS needs to be much lower than that for the silicon substrate in order to characterize the temperature rise difference of the test line between the silicon and the porous silicon. The thickness has also to be high enough in order to influence the thermal dissipation and hence the thermal characterization.

### Conclusions

In this study, we have characterized test lines under a probe station by measuring the resistance rise versus the applied current. This characterization has been performed on two different substrates: a silicon substrate and a mesoporous silicon/silicon substrate. Firstly, the model simulator has been calibrated in order to replicate the experiments, and secondly, the thermal conductivity of the porous silicon layer has been evaluated. The determined value of 4.2 W/(K·m) is in agreement with the value obtained from micro-Raman scattering measurements. Our method appears to be efficient and useful in order to determine thermal conductivity of porous silicon morphology provided that its thermal impact is significant.

### Competing interests

The authors declare that they have no competing interests.

### Authors’ contributions

LS wrote the manuscript and performed electrical characterizations. LS and FR performed electrothermal simulation. MC anodized the silicon substrate. VL performed thermal conductivity micro-Raman measurement. GG participated in the concept of the study and revised the manuscript for important intellectual contents. All authors read and approved the final manuscript.
